# Development of a Patient-Centered Outcome Tool for Blepharospasm: A Stepwise Modified Delphi Study

**DOI:** 10.3390/toxins17090455

**Published:** 2025-09-10

**Authors:** Brian D. Berman, Fares Qeadan, Amanda D. Henderson, Andrew R. Harrison, Giovanni Defazio, Mark Hallett, Gamze Kilic-Berkmen, Laura Wright, Samantha Pentecost, Paul Reyes, Anna Tingin, Joseph Jankovic, Jane Boyd, Charlene Hudgins, Janet Hieshetter, Joel S. Perlmutter, Hyder A. Jinnah, Sarah Pirio Richardson

**Affiliations:** 1 Department of Neurology, Virginia Commonwealth University, Richmond, VA 23298, USA; 2 Department of Public Health Sciences, Loyola University Chicago, Chicago, IL 60153, USA; fqeadan@luc.edu; 3 Department of Ophthalmology, Wilmer Eye Institute, Johns Hopkins University School of Medicine, Baltimore, MD 21287, USA; 4 Departments of Ophthalmology and Otolaryngology, University of Minnesota, Minneapolis, MN 55415, USA; 5 Department of Translational Biomedicine and Neuroscience, Aldo Moro University of Bari, 70124 Bari, Italy; 6 NINDS Intramural Program, Bethesda, MD 20892, USA; 7 Department of Neurology, Emory University School of Medicine, Atlanta, GA 30322, USA; 8 Department of Neurology, Washington University School of Medicine, St. Louis, MO 63110, USAperlmutterjoel@wustl.edu (J.S.P.); 9 Department of Neurology, University of New Mexico Health Sciences Center, Albuquerque, NM 87106, USA; sjpentecost1@salud.unm.edu (S.P.); atingin@salud.unm.edu (A.T.);; 10 Parkinson’s Disease Center and Movement Disorders Clinic, Department of Neurology, Baylor College of Medicine, Houston, TX 77030, USA; 11 Benign Essential Blepharospasm Research Foundation, Beaumont, TX 77726, USAcharlene@blepharospasm.org (C.H.); 12 Dystonia Medical Research Foundation, Chicago, IL 60601, USA

**Keywords:** blepharospasm, patient-centered outcomes, botulinum neurotoxin, self-assessments, symptom monitoring, treatment tracking

## Abstract

Blepharospasm (BSP) is characterized by excessive orbicularis oculi muscle activity leading to abnormal blinking and involuntary eyelid closure. Botulinum neurotoxin (BoNT) injections are the main treatment for BSP, but they only partially and transiently relieve symptoms, leading to a waxing and waning therapeutic response. A patient-centered outcome (PCO) tool that measures BSP symptoms in a simple and efficient way could inform the development of better treatments. Using a stepwise modified Delphi approach, potential PCO items were first identified using the Dystonia Coalition Database with data from over 200 individuals with BSP who had provided responses to existing clinical assessment scales. These items were then analyzed for contribution to overall severity using a Random Forests approach, and redundant items were merged and revised in a series of iterative meetings with a specialist panel along with input from patient advocacy group representatives and focus groups. An online survey was conducted with 330 individuals with BSP to validate and verify the items’ relevance. Finally, the specialist panel provided content validity ratio, which was repeated until it showed good agreement for relevance and clarity of all items. In the end, an easy-to-use PCO tool designed for smartphones and tablets containing 17 items covering three symptom domains (motor, disability, and psychosocial/quality of life) was created. This novel PCO tool for BSP may be used to characterize the cyclical response that an individual patient experiences from BoNT treatments and provide a vital tool for future investigations of longer-acting BoNT preparations or adjunctive therapies.

## 1. Introduction

Blepharospasm (BSP) is a focal dystonia characterized by excessive orbicularis oculi muscle activity leading to abnormal blinking, involuntary eyelid closure, and difficulty with eye opening [1,2]. The prevalence of BSP varies by geographical region but has been estimated to affect up to 130 per million people worldwide [3]. Those with BSP frequently experience spread of dystonia to muscles beyond the orbicularis oculi with approximately half of affected individuals experiencing spread to other muscles such as the lower face and jaw [4,5,6]. BSP is also often associated with sensitivity to bright lights or wind, irritating sensations in the eyes such as dryness or grittiness, impaired activities of daily living, psychosocial difficulties, and diminished quality of life [7]. Furthermore, psychiatric symptoms including depression and anxiety frequently occur with BSP [8,9], which can further impact quality of life [10].

Presently, intramuscular injection of botulinum neurotoxin (BoNT) into the orbicularis oculi and other affected muscles remains the treatment of choice for BSP as no current oral medication provides adequate relief [11]. BoNT treatments generally continue for life because BSP remains incurable and rarely remits. While BoNT injections improve the motor symptoms of BSP and quality of life, they frequently only partially relieve symptoms and they can be painful, wear off quickly, or induce intolerable weakness or other adverse effects. Many BSP individuals are treated at a fixed interval of 12 weeks, despite several studies showing considerable inter-individual differences in duration of effect and repeated calls for more flexible dosing intervals based on individual needs [12,13]. One study showed that nearly half of all individuals with BSP preferred a treatment interval < 12 weeks, while some preferred an interval > 16 weeks [14]. The cyclical pattern of waxing and waning symptoms also occurs with BoNT treatment of cervical dystonia, where the phenomenon has been called the *roller-coaster* [15] or *yo-yo* effect [16]. More clinical trials with newer BoNT formulations or effective adjunctive medications are desperately needed to accelerate the development of new and more effective treatments for BSP.

Treatment satisfaction in BSP could be improved by customizing treatment intervals according to individual needs, developing novel BoNTs with longer duration of action and less prominent fluctuations over the treatment cycle, or developing novel adjunctive oral medications to mitigate symptoms that emerge when BoNT wears off [17,18]. Customizing treatment intervals according to individual needs requires methods to assess efficacy during these treatment intervals, which currently rely almost exclusively on patient recollection at the time of a repeat treatment; however, tools for measuring duration of action in a clinically meaningful way have not been available for BSP. To advance therapeutic treatment options for BSP, more precise temporal information is needed regarding the associated disability and psychiatric symptoms that may or may not align with the magnitude of motor benefit from BoNT.

A tool to measure the temporal effects of BoNT on motor and non-motor symptoms, disability, and quality of life in BSP must be simple and efficient, feasible to use on a weekly basis, and sensitive to changes with relevant treatment interventions. Clinician-rated scales such as the Jankovic Rating Scale [19] or the Defazio Blepharospasm Severity Rating Scale [20] are not suitable for this purpose, because both require in-person assessment by trained clinicians. The Blepharospasm Disability Index (BSDI) [21] has several items (such as driving a car) that assess disability from motor symptoms but may not change in response to short-term interventions [18]. The Craniocervical Dystonia Questionnaire (CDQ-24) [22] can be used to assess quality of life in individuals with BSP, but it is cumbersome for repeated uses and has too many items not relevant to individuals with BSP alone. A patient-centered outcome (PCO) measure that does not require direct assessment by a clinician and can be implemented on a handheld device could provide a detailed assessment of cycling motor and non-motor symptoms in BSP patients receiving BoNT treatment and identify gaps in therapy for novel therapies.

The goal of this study was to develop a pragmatic PCO that incorporates the unique and specific motor and non-motor features affecting those with BSP and that could similarly be easily administered through a digital platform such as a smartphone or tablet. A major advantage of this approach is that real-time data may be collected in the natural environment of individuals with BSP. This method of frequent data sampling (ecological momentary assessment) will enable the generation of a rich source of information for individual subjects that allows for a fuller appreciation of the contributions of different elements to the overall neurological disorder.

## 2. Materials and Methods

### 2.1. Study Design

The PCO for BSP was designed following U.S. Food and Drug Administration (FDA) guidelines for development of novel patient-centric measurement tools that incorporate input from all relevant stakeholders (FDA Guidance for Industry: Patient-Reported Outcome Measures: Use in Medical Product Development to Support Labeling Claims, 2009). Participant data included in this study were collected through the Dystonia Coalition Projects-3: Natural History; Objective Measures; Biobank; Patient-Centered Outcomes (https://dc.rarediseasesnetwork.org/research-study/6305, accessed on 12 August 2025). Inclusion criteria included the diagnosis of isolated BSP, age 18 years or older, fluency in English, ability to complete questionnaires on a smartphone, and stable treatment with BoNT. Exclusion criteria included evidence for acquired dystonia or complex patterns of dystonia, known resistance or atypical responses to BoNT, receiving of BoNT injections to body regions beyond the upper face, and treatment with deep brain stimulation. Institutional ethical standards committee on human experimentation approval was obtained prior to study initiation at each participating site (https://dc.rarediseasesnetwork.org/clinical-sites, accessed on 12 August 2025).

Content development involved a conceptually driven iterative process with three stages followed by a performance testing stage that paralleled the development of a PCO for cervical dystonia [23] and integrated input from clinicians with expertise in treating BSP (movement disorders-trained neurologists and ophthalmologists), patient advocacy groups, and patients with BSP. As BSP often co-occurs with cervical dystonia, one overall objective was to create a PCO that aligns well with a PCO recently created for cervical dystonia [23]. As such, items were grouped into a similarly structured set of three main clinical domains relevant to individuals with adult-onset focal dystonia that can potentially respond to therapeutic intervention (MOTOR, DISABILITY, and PSYCHOSOCIAL). While the number of final items included in the PCO was not strictly defined, another overall objective was to have approximately five final items per domain to balance sufficient detail within a domain and not create too burdensome of a PCO tool that could be answered on a frequent time period (i.e., weekly).

#### 2.1.1. Stage 1: Content Development and Item Generation

The first step was generation of content and specific items. Items were collected from previously used clinical assessment tools such as symptom severity questionnaires used in prior studies of BSP [24], the Blepharospasm Disability Index (BSDI) [21], the Craniocervical Dystonia Questionnaire-24 (CDQ-24) [22], the Beck Depression Inventory II (BDI-II) [25], and the Rand 36-Item Short Form Survey Instrument (SF-36) [26]. Items were categorized into three domains: MOTOR, which included BSP-related motor symptomatology, DISABILITY, which focused on the impact of symptoms on activities of daily living, and PSYCHOSOCIAL, which included mental well-being and health-related quality of life across physical and mental health domains. Items not felt to be suitable for the PCO, such as questions pertaining to regions outside of interest for BSP patients like the fingers, and items not responsive to short-term changes, were eliminated or modified, while similar or overlapping items were merged. Each question was considered to be of equal value and the sum of all values was the Total Score.

Random Forest analysis was performed to identify items that significantly impacted the Total Score across the three domains. This is an ensemble learning technique with robust handling of high-dimensional data, along with the construction of multiple decision trees during training [27]. The mean prediction of each tree was identified for multiple linear regression analysis, aiding the recognition of the relative importance of each item. The significance of items in the Random Forest regression models was determined via an impact on out-of-bag mean square error, a widely used measure of model accuracy that estimates prediction error using unseen data [28]. Variables yielding larger out-of-bag mean square error values were considered more influential, as their removal led to substantial increases in prediction error, indicating their importance in predicting the total score. This strategy helped to prioritize variables to include, and it ensured effectiveness in capturing nuances influencing BSP severity and impact.

#### 2.1.2. Stage 2: Item Improvement and Revision of Items

The second step included revision and improvement of each item. This iterative process began with a panel of clinical specialists (movement disorders-trained neurologists and ophthalmologists with extensive expertise with BSP), as well as patient advocacy group representatives from the Benign Essential Blepharospasm Research Foundation (https://blepharospasm.org, accessed on 12 August 2025) and the Dystonia Medical Research Foundation (https://dystonia-foundation.org, accessed on 12 August 2025). The panel assessed all candidate items to determine if key items were missing, and they ranked all according to perceived level of importance. The ranking involved a 9-point Likert scale ranging from not important (1) to critically important (9). The final candidate list was reduced to the most important items.

Candidate items identified by the specialist panel were then refined via three teleconferences with stakeholder focus groups held approximately one week apart with ~10 individuals with BSP per focus group. The objectives given to the focus groups were to rank the items across the three domains according to the personal perspective of individuals with BSP, help improve the clarity of any confusing items, and to identify any important missing or overlooked items. Qualitative analyses were carried out following the principles of thematic narrative analysis to identify common themes found through participant feedback [29]. Qualitative data were evaluated by counting thematic categories of coded responses for each of the items.

Candidate items incorporated all feedback from the focus groups, and then the chair of the BSP specialist panel (BDB) worked with the chair of the cervical dystonia specialist panel (SPR) to make a set of minor modifications to help harmonize the language structure of DISABILITY and PSYCHOSOCIAL items with overlapping items in the recently developed PCO tool for cervical dystonia. Harmonization included matching the language used for comparable symptoms across dystonia types to avoid overlapping and redundant questions should the BSP PCO be combined with the PCO being developed for CD. While the current instrument is not tailored for patients with both BSP and CD, future opportunities could include combined PCO assessments for those with multiple affected areas by dystonia. After this step, the finalized candidate items were evaluated in a content validity stage.

#### 2.1.3. Stage 3: Content Validity

The quantification of content validity rating (CVR) process was used to assess content validity [30]. All members of the BSP specialist panel rated each candidate item for degree of RELEVANCY and CLARITY using a 4-point scale: 1 for “not relevant/clear”, 2 for “item needs some revision, 3 for “relevant/clear but needs minor revision,” and 4 for “very relevant/clear.” After compiling results from all members of the panel, any items with CVR < 0.62 were revised and re-assessed until consensus was reached [30]. At this stage, rating anchors for each item were selected (e.g., “Never” to “Always” or “None” to “Extreme.”)

Next, an online patient survey was conducted to collect additional feedback from a large population sample of individuals with BSP. The survey was advertised through patient advocacy groups, and any affected individual could participate. The survey began with a screening question asking the participant to self-report if they had a diagnosis of BSP. Individuals who responded affirmatively were then asked to read the instructions and answer all subsequent survey questions. Participants were then presented with each PCO domain item and asked three questions: (1) “Does this item reflect your experience with blepharospasm?”, (2) “Would a treatment that can improve this symptom be meaningful to you?”, and (3) “What minimal amount of improvement would be meaningful to you (ranging from 0% for no improvement to 100% for full improvement)?”.

Responses to the third question were analyzed to determine the Minimal Meaningful Improvement threshold for each symptom. Descriptive statistics, including medians, quartiles, and percentiles, were calculated to assess the distribution of responses. Clinically relevant thresholds were established by defining small, medium, and large effect sizes based on cumulative response distributions. A small effect size was defined as the minimum improvement percentage at which at least 95% of respondents considered the change meaningful, a medium effect size was determined at the 90% threshold, and a large effect size was defined as the improvement level reported by at least 75% of participants. An additional exploratory threshold of 50% was applied to identify substantial perceived benefit. These calculations were conducted using SAS v9.4 software, and results were analyzed separately for each symptom domain (MOTOR, DISABILITY, and PSYCHOSOCIAL) to account for variations in symptom burden and patient-reported impact.

#### 2.1.4. Stage 4: Testing Performance of PCO

The performance of the PCO for capturing changes in the severity of individual items at weekly intervals over a single BoNT treatment cycle was assessed for five individuals with BSP. Final PCO items from Stage 3 were programmed into an application (SymptomSnap, developer: TekSynap Corporation, Reston, VA, USA, all rights reserved) and downloaded on the participant’s smartphone or tablet. Responses for all PCO items were collected using a sliding scale from 0 to10 to rate each item between the anchors. An automatic reminder to enter ratings was sent to the participant’s mobile device once every week. After 12 weeks, each individual was asked three additional survey questions regarding usability including: (1) “How easy was this tool to use?”, (2) “Do you think it is useful to document changes in the severity of your condition?”, and (3) “Would you recommend this tool to others?”.

## 3. Results

**Figure 1:**
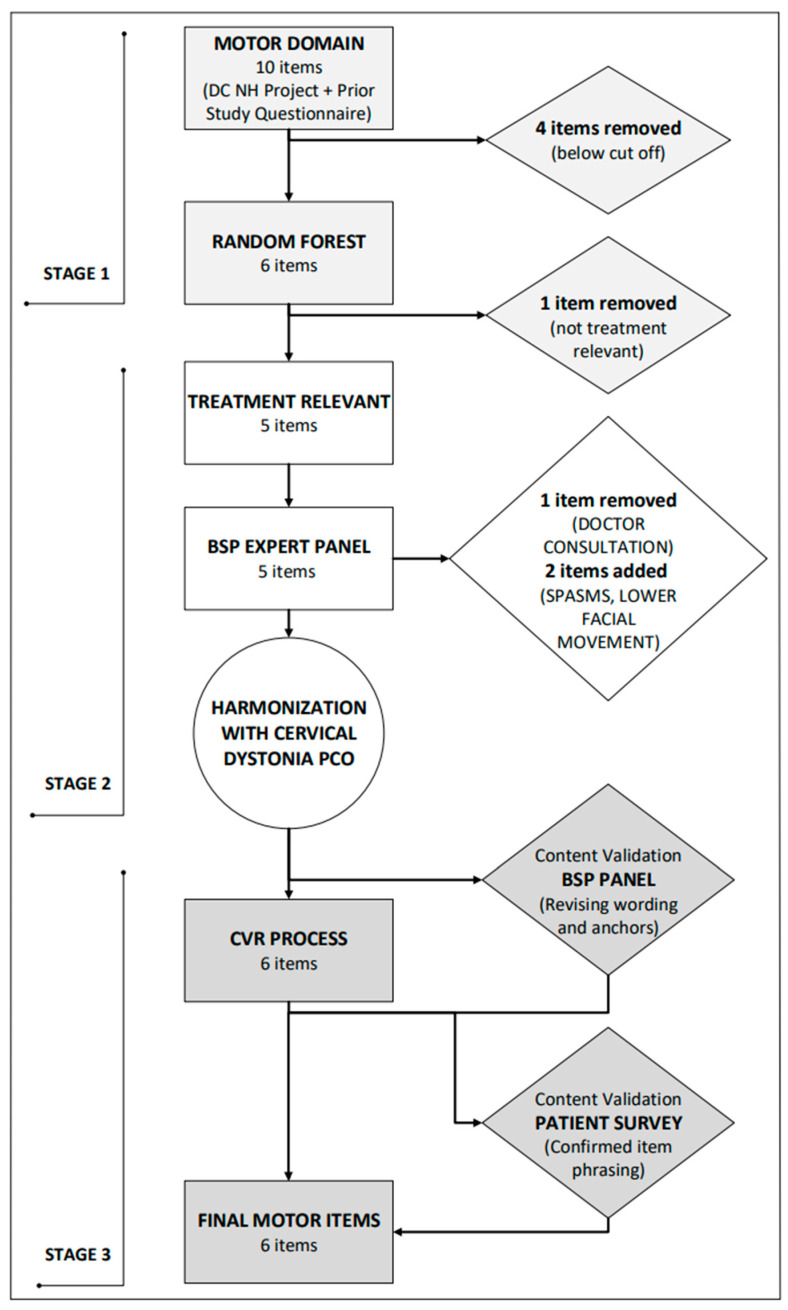
Flow diagram for the development of the blepharospasm (BSP) patient-centered outcome (PCO)—MOTOR domain. Item selection, revision, and finalization went through three stages of the process for the MOTOR domain (see Supplemental Materials for flow diagrams of DISABILITY and PSYCHOSOCIAL domains). Stage 1 involved content development and item generation, Stage 2 was focused on item improvement and revision of items, and Stage 3 involved a content validity rating (CVR) process to ensure content validity.

As shown in Figure 1, the content development and item generation stage yielded 10 items within the MOTOR domain subsequently reduced to 5 items. A similar process was used to reduce 30 items within the DISABILITY domain to 11 items (Figure S1A) and 63 items within the PSYCHOSOCIAL domain to 16 items (Figure S1B). Items were subsequently removed after Random Forest regression and due to duplication or lack of expected response to treatment interventions. All items included wording emphasizing the relevance of the question to BSP, to avoid answering in a way that reflected other comorbidities. Given that about half of those with BSP will develop involvement of the oromandibular region, an item was added to the MOTOR domain to try and capture the severity of lower face (tongue, mouth, and jaw) dystonia. In addition, items were added to the DISABILITY and PSYCHOSOCIAL domains to capture the impact of oromandibular involvement on these domains.

At the content validity rating stage, item wording and anchors were revised. With several iterations of the CVR process within the specialist panel, all final items were above the cut-off for clarity and relevancy (see Table 1). In Table 1, only the medium effect size (90% threshold) is reported to provide a standardized measure of meaningful improvement across symptoms. Relevance was then tested in the broader dystonia community using an online patient survey. The survey was advertised via email by the Benign Essential Blepharospasm Research Foundation and Dystonia Medical Research Foundation. The survey remained open for four weeks, and during this time 330 unique responses were collected. All items had a concurrence rate higher than 70% for relevancy with the exception of the three questions related to oromandibular muscle involvement (Table 1). The decreased relevancy for the lower face involvement is expected given that only about half of patients with BSP have oromandibular involvement.

As shown in Table 1, the items with best concurrence were questions on difficulty “keeping your eyes fully open” and whether spasms cause eyes to close “against your will”, both endorsed by 94% of respondents. In contrast, the item with the lowest concurrence was whether there was limitation in “talking and/or eating”, which was endorsed by only 34% of participants. There was substantial variability in the desired level of improvement among individuals with BSP. The largest minimal meaningful improvement threshold was observed for driving limitations (42%), while the smallest meaningful improvement threshold was reported for talking and/or eating limitations (23%) (Table 1). Regarding effect size thresholds, at least 95% of respondents reported that a 20% improvement (SD 6.1%) was the smallest meaningful change (small effect size). At least 90% of respondents required a 31% improvement (SD 5.8%) for the change to be considered clinically significant (medium effect size), while at least 75% of respondents required a 49% improvement (SD 3.1%) to consider the change meaningful (large effect size). Additionally, 50% of respondents identified a 61% improvement (SD 6.5%) as the threshold for substantial perceived benefit.

**Figure 2:**
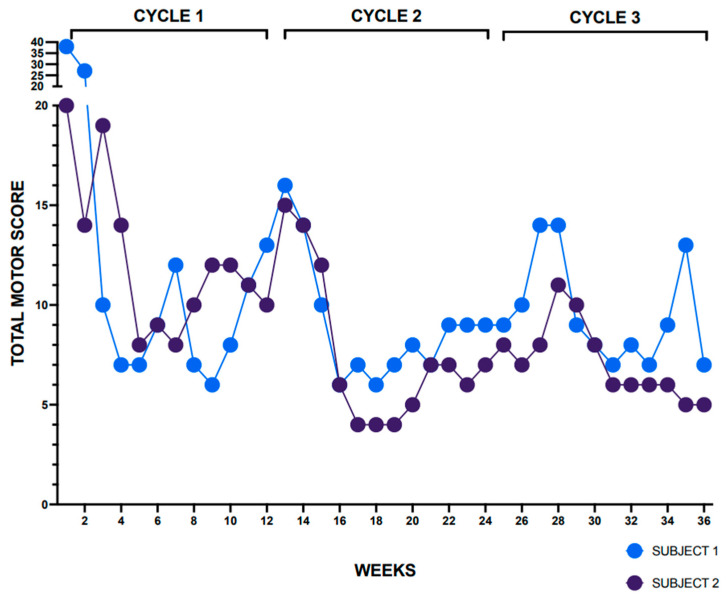
Patient-centered outcome total MOTOR scores for two randomly selected individuals with blepharospasm. Scores were obtained weekly over three 12-week botulinum neurotoxin treatment (BoNT) treatment cycles. The total MOTOR scores for both individuals decrease after the BoNT treatment cycles, with the greatest benefit observed around 6–8 weeks after the injection and wearing-off of treatment effect observed around 10–12 weeks after the injection.

To assess real-world performance in a pilot project, the PCO was made available on the SymptomSnap app to five individuals with BSP. Each participant was asked to record responses weekly over a single BoNT injection cycle and of those five, two individuals with BSP were asked to record responses weekly over three complete BoNT cycles (Figure 2). These participants showed good adherence with weekly assessments with less than 5.0% missing data. They further reported that the app was easy to use and required less than 10 min to enter symptoms, and that they would recommend the app to others. As can be seen in Figure 2, the total PCO MOTOR scores for the two individuals followed for three BoNT cycles followed an expected “yo-yo” pattern with subjective improvement in motor symptoms after each injection and waning of benefit prior to the next BoNT injection. The patterns of the two pilot participants further revealed that their perceived total motor symptom severity tends to vary from week to week.

**Figure 3:**
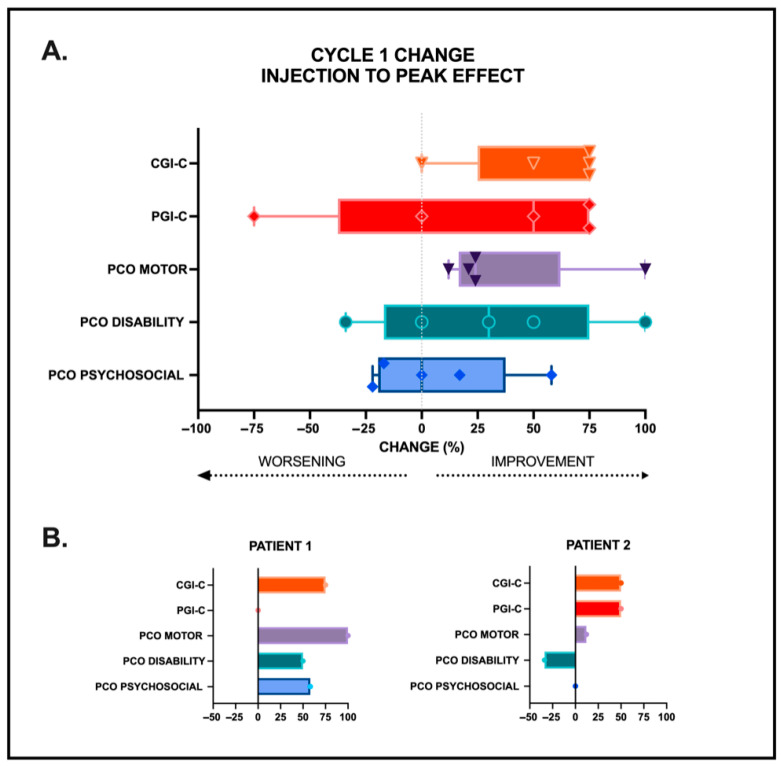
Percent change from assessments at time of botulinum neurotoxin (BoNT) injections to peak effect (typically 6–8 weeks after injection). Results are shown for five individuals in a pilot project assessment (**A**) with data from two individuals shown separately (**B**) to highlight individual differences across the different rating measures. Change in the positive direction indicates improvement in symptoms from injection to peak effect. Congruence largely seen with clinician ratings (CGI-C: Clinical Global Impression of change) and patient ratings (PGI-C: Patient Global Impression of change) as well as with total patient-centered outcome (PCO) MOTOR domain score change. Less congruence was seen with percent changes in the total PCO DISABILITY and PSYCHOSOCIAL domain scores.

The precent changes in the Global Impression of change scores rated by clinician (CGI-C) and patient (PGI-C) along with the precent changes in the PCO scores from peak effect to starting baseline BoNT injection of the five BSP participants followed over a single 12-week cycle are plotted in Figure 3A. Interestingly, one participant reported a worsening in motor symptoms in this small pilot sample. Nevertheless, the data reveal general congruence of motor severity as measured by the CGI-C, PGI-C, and PCO MOTOR scores. When looking at the responses of two representative patients (Figure 3B), it is apparent that while subjective motor improvement following BoNT injections tended to align with clinician ratings of dystonia improvement (PGI-C), the PCO-based disability and psychosocial/quality of life changes may not align. This section may be divided by subheadings. It should provide a concise and precise description of the experimental results, their interpretation, as well as the experimental conclusions that can be drawn.

## 4. Discussion

Described here is the development of an easy-to-use digital tool for gathering information at frequent intervals regarding changes in the motor and non-motor symptoms that are most meaningful to individuals with BSP, and preliminary results demonstrating the feasibility of this novel PCO tool are provided. The app-based tool can be used to collect data at any interval, with good adherence observed using weekly intervals in a small pilot. The weekly collection interval was sufficient to document the temporal profile of responses of individual symptoms in response to BoNT over three 12-week treatment cycles, as shown in the pilot data presented here.

A critical aspect of this study was the rigorous statistical methodology employed to refine and validate the PCO tool. The application of Random Forest regression, a widely used machine learning technique for high-dimensional data analysis, allowed for an objective, data-driven selection of the most relevant items while eliminating redundancies [27]. This approach has been increasingly applied in healthcare and clinical research for optimizing patient-reported outcome measures [31,32], and it ensures that the final tool maintained high predictive validity and retained the most meaningful symptom descriptors. Furthermore, the iterative involvement of both clinical experts and patient advocacy groups through multiple rounds of validation aligns with best practices in PCO measure development as recommended by the US Food and Drug Administration and the Patient Centered Outcomes Research Institute [33]. This approach maximizes the relevance and applicability of the tool, ensuring that it accurately captures the most clinically and patient-relevant symptoms.

The minimum clinically important difference (MCID) in a clinical rating is often defined as the smallest change in a patient-reported outcome measure that a patient would perceive as beneficial, such as a patient global impression of change rating or a quality-of-life measure (e.g., SF-36) [34,35]. In the current study, the MCID was obtained by directly asking patients about each of the PCO symptom items deemed most relevant, a method aligned with best practices in PCO-based MCID estimation [36,37]. An interesting finding that emerged from this study is that the degree of desired improvement was symptom-specific rather than uniform across all domains. This is expected, as some BSP symptoms are more intrusive than others. In particular, the statistical analysis highlighted variability in the thresholds of desired meaningful improvement across PCO items and symptom domains. Motor symptoms such as difficulty keeping the eyes open and involuntary spasms were associated with higher thresholds for meaningful improvement (40% and 37%, respectively), whereas symptoms of anxiousness and limitation in social situations due to the eye problem had lower thresholds (27% and 26%, respectively). This finding suggests that a standardized interpretation of PCOs may not be appropriate as different symptoms carry different burdens for individual patients. Future studies should therefore consider a symptom-specific approach when assessing treatment efficacy and defining clinical benchmarks, rather than relying on a single global MCID threshold [38,39].

Presently, prospective BSP PCO data are being collected in a multi-site study through the Dystonia Coalition that will enable a formal analysis of data acquired with the PCO tool and SymptomSnap app in a large cohort and over multiple usual care BoNT cycles. This newly developed PCO measure in BSP could have several applications. First, it has immediate practical value for ongoing BoNT treatment, serving as a medical symptom diary, similar to the digital PCO recently developed for cervical dystonia that was also found to be sensitive to short-term changes in motor and non-motor symptomatology [23]. The PCO could help clinicians better understand an individual patient’s response to BoNT injections, as real-time data collection is likely more reliable than relying on patient recall at follow-up visits. The development of a valid app-based PCO tool in BSP could further inform the development of PCOs for other adult-onset focal dystonia subtypes such as hand and laryngeal dystonia and help guide BoNT and rehabilitative strategies aimed at improving motor performance and reducing disability [40,41,42]. Additionally, these data could also be used to inform dose and/or treatment interval adjustments to optimize the treatment outcomes. Third, the PCO tool could be used to chart the temporal dynamics of one or more BoNT treatment cycles, permitting assessment of symptom responses to novel BoNT formulations that may have longer durations of effect or reduced symptom fluctuations compared to existing formulations. Finally, the PCO could serve as a valuable instrument for documenting the potential benefit of adjunct oral therapies across one or more BoNT treatment cycles.

Limitations of the present study include the potential for steering group bias in the selection of the expert panels used for the modified Delphi approach, as well as the potential consequences stemming from reduced anonymity in both the expert panels and patient groups. Limited iterative rounds could have also affected the robustness of the modified Delphi method. Another limitation of our study is that the number of overall items to be included in the final PCO tool, while not strictly defined, were limited in overall number so there are likely to be motor and non-motor symptoms and quality of life measures affecting some with BSP that have not been included. This was a necessary tradeoff in order to be able to collect more frequent assessments and not create a PCO tool that was felt to be too burdensome to complete by patients.

## 5. Conclusions and Recommendations

In conclusion, an app-based PCO tool was developed for BSP that enables the capturing in real-time of motor and non-motor symptom fluctuations that commonly occur during routine BoNT treatment cycles. The PCO was designed to enable the frequent collection of severity information for the most relevant symptoms affecting those with BSP in a non-burdensome manner. The use of this PCO tool can easily be integrated into clinical trials and has the potential to enhance the evaluation of novel combination therapies, ultimately guiding future treatment advancements for BSP. The user-friendliness of the app and these applications gives the PCO tool a distinct advantage over self-administered clinical rating scales in future clinical trials.

## Figures and Tables

**Table 1:** Patient-centered outcome items evaluated for content validity by specialist panels and for relevancy by patient panels.

DOMAIN	ITEMS	Content Validity Ratio		Patient Concurrence *(Item Relevancy, %)*	Desired Improvement Threshold *(90th Percentile)*
		*Relevancy*	*Clarity*		
**MOTOR**	How often do you blink too much?	1.0	0.71	83	40
	How much difficulty do you have keeping your eyes fully open?	1.0	1.0	94	40
	How much discomfort do you have with bright light?	0.86	0.86	90	35
	How much discomfort do you have due to any gritty, sandy or burning sensations in your eyes?	0.86	1.0	74	30
	Do spasms close your eyes against your will?	1.0	1.0	94	37
	How often do you experience uncontrollable movements of your tongue, mouth, or jaw?	1.0	1.0	43	25
**DISABILITY**	How much limitation do you have in work performance (either household work or outside employment) due to your eye or mouth problem?	1.0	1.0	77	28
	How much limitation do you have in driving due to your eye or mouth problem?	1.0	1.0	83	42
	How much limitation do you have in your leisure activities due to your eye or mouth problem?	0.86	0.86	86	30
	How much limitation do you have in talking and/or eating due to uncontrollable movements of your tongue, mouth, or jaw?	1.0	1.0	34	23
**PSYCHOSOCIAL**	How often do you feel anxious due to your eye or mouth problem?	1.0	1.0	86	27
	How often do you feel down or depressed due to your eye or mouth problem?	1.0	1.0	73	27
	How often do you feel frustrated due to your eye or mouth problem?	0.86	1.0	93	30
	How often do you feel embarrassed due to your eye or mouth problem?	1.0	1.0	81	27
	How much limitation do you have in social situations (e.g., visiting friends and family, attending events outside the home, etc.) due to your eye problem?	0.86	0.71	84	26
	How much limitation do you have in social situations (e.g., visiting friends and family, attending events outside the home, etc.) due to uncontrollable movements of your tongue, mouth, or jaw?	0.86	0.71	41	30
	How much is your quality of life affected due to your eye or mouth problem?	1.0	1.0	92	30

## Data Availability

Data that were used in the generation of our patient-centered outcome tool are publicly available from the Dystonia Coalition through a data request. Requests to access the datasets should be directed to https://dc.rarediseasesnetwork.org/resources-researchers-and-clinicians, accessed on 12 August 2025.

## Supplementary Materials

The following supporting information can be downloaded at: https://www.mdpi.com/article/10.3390/toxins17090455/s1, Figure S1. Flow diagram of our blepharospasm (BSP) patient-centered outcome (PCO) item selection, revision, and finalization through the three stages of the process for the DISABILITY and PSYCHOSOCIAL domains.

## Abbreviations

The following abbreviations are used in this manuscript:

BSP	Blepharospasm
BoNT	Botulinum neurotoxin
BSDI	Blepharospasm Disability Index
CDQ-24	Craniocervical Dystonia Questionnaire
PCO	Patient-centered outcome
CVR	Content validity rating
SAS	Statistical Analysis Software
CGI-C	Clinical Global Impression-Clinician
CGI-P	Clinical Global Impression-Patient
MCID	Minimum clinically important difference

## References

[1] Defazio G., Jinnah H.A., Berardelli A., Perlmutter J.S., Berkmen G.K., Berman B.D., Jankovic J., Bäumer T., Comella C., Cotton A.C. (2021). Diagnostic Criteria for Blepharospasm: A Multicenter International Study. Park. Relat. Disord..

[2] Scorr L.M., Cho H.J., Kilic-Berkmen G., McKay J.L., Hallett M., Klein C., Baumer T., Berman B.D., Feuerstein J.S., Perlmutter J.S. (2022). Clinical Features and Evolution of Blepharospasm: A Multicenter International Cohort and Systematic Literature Review. Dystonia.

[3] Zhu L., Meng H., Zhang W., Xie W., Sun H., Hou S. (2024). The Pathogenesis of Blepharospasm. Front. Neurol..

[4] Berman B.D., Groth C.L., Sillau S.H., Pirio Richardson S., Norris S.A., Junker J., Brüggemann N., Agarwal P., Barbano R.L., Espay A.J. (2020). Risk of Spread in Adult-Onset Isolated Focal Dystonia: A Prospective International Cohort Study. J. Neurol. Neurosurg. Psychiatry.

[5] Svetel M., Pekmezović T., Jović J., Ivanović N., Dragašević N., Marić J., Kostić V.S. (2007). Spread of Primary Dystonia in Relation to Initially Affected Region. J. Neurol..

[6] Weiss E.M., Hershey T., Karimi M., Racette B., Tabbal S.D., Mink J.W., Paniello R.C., Perlmutter J.S. (2006). Relative Risk of Spread of Symptoms among the Focal Onset Primary Dystonias. Mov. Disord..

[7] Junker J., Hall J., Berman B.D., Vidailhet M., Roze E., Bäumer T., Malaty I.A., Shukla A.W., Jankovic J., Reich S.G. (2024). Longitudinal Predictors of Health-Related Quality of Life in Isolated Dystonia. J. Neurol..

[8] Berman B.D., Junker J., Shelton E., Sillau S.H., Jinnah H.A., Perlmutter J.S., Espay A.J., Jankovic J., Vidailhet M., Bonnet C. (2017). Psychiatric Associations of Adult-Onset Focal Dystonia Phenotypes. J. Neurol. Neurosurg. Psychiatry.

[9] Defazio G., Gigante A.F., Hallett M., Berardelli A., Perlmutter J.S., Berman B.D., Jankovic J., Bäumer T., Comella C., Ercoli T. (2022). Motor and Psychiatric Features in Idiopathic Blepharospasm: A Data-Driven Cluster Analysis. Park. Relat. Disord..

[10] Junker J., Berman B.D., Hall J., Wahba D.W., Brandt V., Perlmutter J.S., Jankovic J., Malaty I.A., Wagle Shukla A., Reich S.G. (2021). Quality of Life in Isolated Dystonia: Non-Motor Manifestations Matter. J. Neurol. Neurosurg. Psychiatry.

[11] Duarte G.S., Rodrigues F.B., Marques R.E., Castelão M., Ferreira J., Sampaio C., Moore A.P., Costa J. (2020). Botulinum Toxin Type A Therapy for Blepharospasm. Cochrane Database Syst. Rev..

[12] Leplow B., Eggebrecht A., Pohl J. (2017). Treatment Satisfaction with Botulinum Toxin: A Comparison between Blepharospasm and Cervical Dystonia. Patient Prefer Adherence.

[13] Ojo O.O., Fernandez H.H. (2015). Is It Time for Flexibility in Botulinum Inter-Injection Intervals?. Toxicon.

[14] Fezza J., Burns J., Woodward J., Truong D., Hedges T., Verma A. (2016). A Cross-Sectional Structured Survey of Patients Receiving Botulinum Toxin Type A Treatment for Blepharospasm. J. Neurol. Sci..

[15] Comella C., Ferreira J.J., Pain E., Azoulai M., Om S. (2021). Patient Perspectives on the Therapeutic Profile of Botulinum Neurotoxin Type A in Cervical Dystonia. J. Neurol..

[16] Pirio Richardson S., Jinnah H.A. (2019). New Approaches to Discovering Drugs That Treat Dystonia. Expert Opin. Drug Discov..

[17] Comella C.L., Jankovic J., Hauser R.A., Patel A.T., Banach M.D., Ehler E., Vitarella D., Rubio R.G., Gross T.M., on behalf of the ASPEN-1 Study Group (2024). Efficacy and Safety of DaxibotulinumtoxinA for Injection in Cervical Dystonia: ASPEN-1 Phase 3 Randomized Controlled Trial. Neurology.

[18] Kilic-Berkmen G., Kim H., Chen D., Yeo C.I., Dinasarapu A.R., Scorr L.M., Yeo W., Peterson D.A., Williams H., Ruby A. (2024). An Exploratory, Randomized, Double-Blind Clinical Trial of Dipraglurant for Blepharospasm. Mov. Disord..

[19] Jankovic J., Kenney C., Grafe S., Goertelmeyer R., Comes G. (2009). Relationship between Various Clinical Outcome Assessments in Patients with Blepharospasm. Mov. Disord..

[20] Defazio G., Hallett M., Jinnah H.A., Stebbins G.T., Gigante A.F., Ferrazzano G., Conte A., Fabbrini G., Berardelli A. (2015). Development and Validation of a Clinical Scale for Rating the Severity of Blepharospasm. Mov. Disord..

[21] Defazio G., Hallett M., Berardelli A., Perlmutter J.S., Berman B.D., Jankovic J., Bäumer T., Comella C., Ercoli T., Ferrazzano G. (2022). Measurement Properties of Clinical Scales Rating the Severity of Blepharospasm: A Multicenter Observational Study. Mov. Disord. Clin. Pract..

[22] Muller J. (2004). Craniocervical Dystonia Questionnaire (CDQ-24): Development and Validation of a Disease-Specific Quality of Life Instrument. J. Neurol. Neurosurg. Psychiatry.

[23] Pirio Richardson S., Berman B.D., Hieshetter J., Comella C., Peterson D.A., Kilic-Berkmen G., Wright L., Pentecost S., Reyes P., Jankovic J. (2024). A Digital Patient-Centered Outcome Tool for Cervical Dystonia. Dystonia.

[24] Martino D., Defazio G., Alessio G., Abbruzzese G., Girlanda P., Tinazzi M., Fabbrini G., Marinelli L., Majorana G., Buccafusca M. (2005). Relationship between Eye Symptoms and Blepharospasm: A Multicenter Case–Control Study. Mov. Disord..

[25] Beck A.T., Steer R.A., Brown G. (1996). Beck Depression Inventory–II 2011.

[26] Jenkinson C., Coulter A., Wright L. (1993). Short Form 36 (SF36) Health Survey Questionnaire: Normative Data for Adults of Working Age. BMJ.

[27] Breiman L. (2001). Random Forests. Mach. Learn..

[28] Strobl C., Boulesteix A.-L., Kneib T., Augustin T., Zeileis A. (2008). Conditional Variable Importance for Random Forests. BMC Bioinform..

[29] Dixon-Woods M., Agarwal S., Jones D., Young B., Sutton A. (2005). Synthesising Qualitative and Quantitative Evidence: A Review of Possible Methods. J. Health Serv. Res. Policy.

[30] O’Keefe-McCarthy S., McGillion M., Nelson S., Clarke S., McFetridge-Durdle J., Watt-Watson J. (2014). Content Validity of the Toronto Pain Management Inventory-Acute Coronary Syndrome Version. Can. J. Cardiovasc. Nurs..

[31] Tschuggnall M., Grote V., Pirchl M., Holzner B., Rumpold G., Fischer M.J. (2021). Machine Learning Approaches to Predict Rehabilitation Success Based on Clinical and Patient-Reported Outcome Measures. Inform. Med. Unlocked.

[32] Verma D., Jansen D., Bach K., Poel M., Mork P.J., d’Hollosy W.O.N. (2022). Exploratory Application of Machine Learning Methods on Patient Reported Data in the Development of Supervised Models for Predicting Outcomes. BMC Med. Inf. Decis. Mak..

[33] Cella D., Hahn E., Jensen S., Butt Z., Nowinski C., Rothrock N., Lohr K. (2015). Patient-Reported Outcomes in Performance Measurement.

[34] Jaeschke R., Singer J., Guyatt G.H. (1989). Measurement of Health Status. Ascertaining the Minimal Clinically Important Difference. Control Clin. Trials.

[35] King M.T. (2011). A Point of Minimal Important Difference (MID): A Critique of Terminology and Methods. Expert Rev. Pharmacoecon. Outcomes Res..

[36] Copay A.G., Subach B.R., Glassman S.D., Polly D.W., Schuler T.C. (2007). Understanding the Minimum Clinically Important Difference: A Review of Concepts and Methods. Spine J..

[37] Revicki D., Hays R.D., Cella D., Sloan J. (2008). Recommended Methods for Determining Responsiveness and Minimally Important Differences for Patient-Reported Outcomes. J. Clin. Epidemiol..

[38] Wyrwich K.W., Norquist J.M., Lenderking W.R., Acaster S. (2013). Industry Advisory Committee of International Society for Quality of Life Research (ISOQOL) Methods for Interpreting Change over Time in Patient-Reported Outcome Measures. Qual. Life Res..

[39] Yost K.J., Eton D.T. (2005). Combining Distribution- and Anchor-Based Approaches to Determine Minimally Important Differences: The FACIT Experience. Eval. Health Prof..

[40] Ashworth N., Aidoo H., Doroshenko A., Antle D., Els C., Flaschner D.M., Gross D.P., Guptill C., Potter P., Tan M.C. (2019). Botulinum Toxin for the Treatment of Focal Task-Specific Hand Dystonias: Systematic Review and Meta-Analysis. Open Neurol. J..

[41] Chiaramonte R., Vecchio M. (2021). Rehabilitation of Focal Hand Dystonia in Musicians: A Systematic Review of the Studies. Rev. Neurol..

[42] Yeung W., Richards A.L., Novakovic D. (2022). Botulinum Neurotoxin Therapy in the Clinical Management of Laryngeal Dystonia. Toxins.

