# Supplementary material for: Development of a Patient-Centered Outcome Tool for Blepharospasm: A Stepwise Modified Delphi Study
Source: Toxins (Basel). 2025 Sep 10;17(9):455. doi: 10.3390/toxins17090455 (PMC12474189; doi:10.3390/toxins17090455)
Supplement: Supplementary file 1 [file toxins-17-00455-s001.zip › toxins-3774588-supplementary.pdf]

FIGURE LEGEND

Supplementary Figure 1. Flow diagram of our blepharospasm (BSP) patient-centered outcome (PCO) item selection, revision, and finalization through the three stages of the process for the DISABILITY and PSYCHOSOCIAL domains. Stage 1 involved content development and item generation, Stage 2 was focused on item improvement and revision of items, and Stage 3 involved a content validity rating (CVR) process to ensure content validity.

Figure S1.

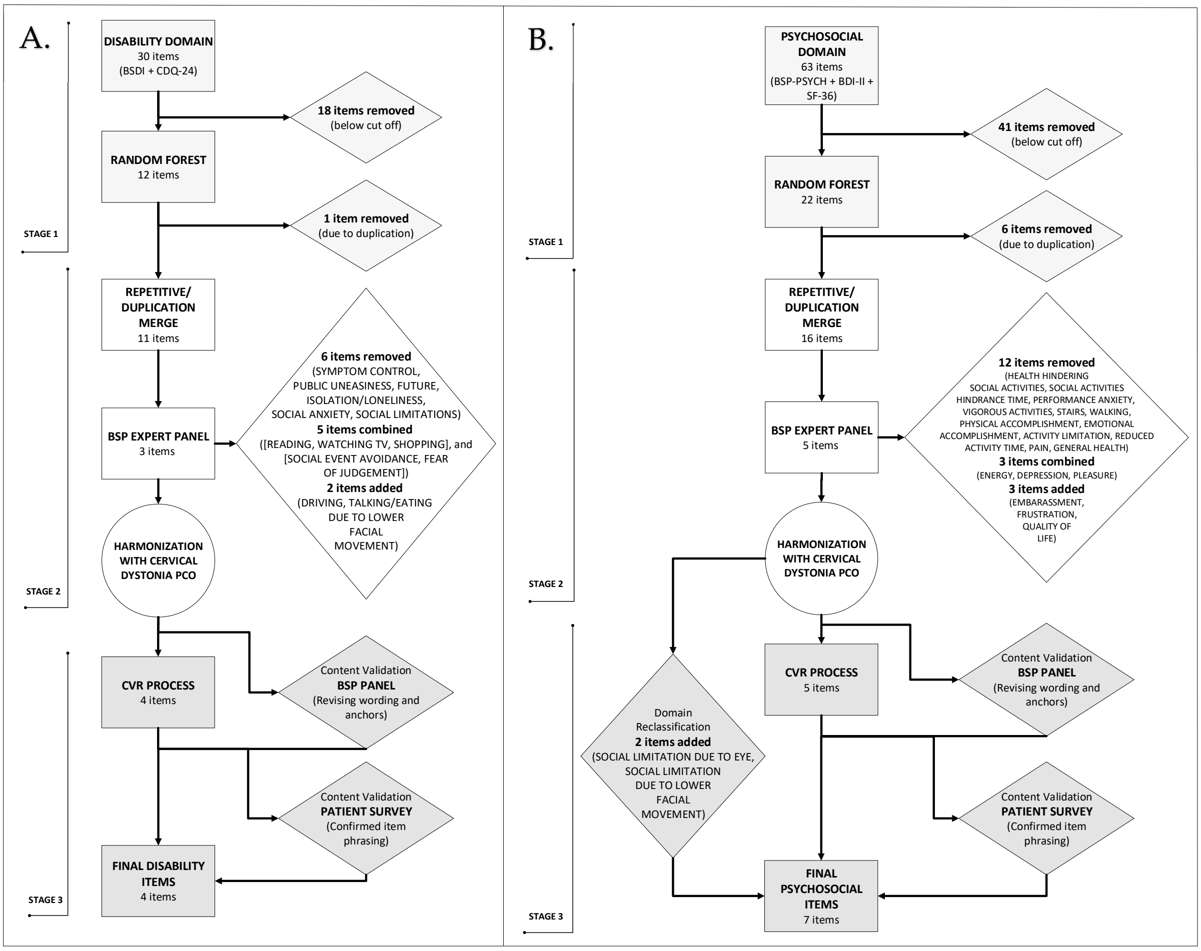
A.
DISABILITY DOMAIN
30 items
(BSDI + CDQ-24)
18 items removed
(below cut off)
RANDOM FOREST
12 items
1 item removed
(due to duplication)
STAGE 1
REPETITIVE/
DUPLICATION
MERGE
11 items
BSP EXPERT PANEL
3 items
6 items removed
(SYMPTOM CONTROL,
PUBLIC UNEASINESS, FUTURE,
ISOLATION/LONELINESS,
SOCIAL ANXIETY, SOCIAL LIMITATIONS)
5 items combined
([READING, WATCHING TV, SHOPPING], and
[SOCIAL EVENT AVOIDANCE, FEAR
OF JUDGEMENT])
2 items added
(DRIVING, TALKING/EATING
DUE TO LOWER
FACIAL
MOVEMENT)
HARMONIZATION
WITH CERVICAL
DYSTONIA PCO
STAGE 2
CVR PROCESS
4 items
Content Validation
BSP PANEL
(Revising wording and
anchors)
Content Validation
PATIENT SURVEY
(Confirmed item
phrasing)
FINAL DISABILITY
ITEMS
4 items
STAGE 3
B.
PSYCHOSOCIAL
DOMAIN
63 items
(BSP-PSYCH + BDI-II +
SF-36)
41 items removed
(below cut off)
RANDOM FOREST
22 items
6 items removed
(due to duplication)
STAGE 1
REPETITIVE/
DUPLICATION
MERGE
16 items
BSP EXPERT PANEL
5 items
12 items removed
(HEALTH HINDERING
SOCIAL ACTIVITIES, SOCIAL ACTIVITIES
HINDRANCE TIME, PERFORMANCE ANXIETY,
VIGOROUS ACTIVITIES, STAIRS, WALKING,
PHYSICAL ACCOMPLISHMENT, EMOTIONAL
ACCOMPLISHMENT, ACTIVITY LIMITATION, REDUCED
ACTIVITY TIME, PAIN, GENERAL HEALTH)
3 items combined
(ENERGY, DEPRESSION, PLEASURE)
3 items added
(EMBARASSMENT,
FRUSTRATION,
QUALITY OF
LIFE)
HARMONIZATION
WITH CERVICAL
DYSTONIA PCO
STAGE 2
CVR PROCESS
5 items
Content Validation
BSP PANEL
(Revising wording and
anchors)
Domain
Reclassification
2 items added
(SOCIAL LIMITATION DUE TO EYE,
SOCIAL LIMITATION
DUE TO LOWER
FACIAL
MOVEMENT)
Content Validation
PATIENT SURVEY
(Confirmed item
phrasing)
FINAL
PSYCHOSOCIAL
ITEMS
7 items
STAGE 3
